# Complexes With Biologically Active Ligands. Part 7^1^ Synthesis and Fungitoxic Activity of Metal Complexes Containing 1,3,5-tris-(8-Hydroxyquinolino)- Trichlorocyclo-Triphosphazatriene

**DOI:** 10.1155/MBD.1996.233

**Published:** 1996

**Authors:** Mihai Barboiu, Cornelia Guran, Ioana Jitaru, Marilena Cimpoesu, Claudiu T. Supuran

**Affiliations:** 1 Research Center for Macromolecular Materials and Membranes Splaiul Independentei 206 Bucharest 79611 Romania; 2 Polytechnic University of Bucharest Department of Inorganic Chemistry Polizu Str. 1 Bucharest 79611 Romania; 3 University of Bucharest Department of Inorganic Chemistry Str. Dumbrava Rosie 23 Bucharest Romania; 4 Università degli Studi Dipartimento di Chimica Laboratorio di Chimica Inorganica e Bioinorganica Via Gino Capponi 7 Florence I-50121 Italy

## Abstract

Complexes containing 1,3,5-tris-(8-hydroxyquinolino)-trichlorocyclotriphosphazatriene, a new cyclophosphazene ligand, and Co(II), Cu(II) and Ni(II) were prepared. The new complexes, having the general formula [MLCl_2_], [ML_2_]Cl_2_, (M=Cu, Co, Ni); [NiLAc], [NiL_2_Ac]Ac and [ML_3_]X_3_ (M=Ni, Co, X=Cl, Ac) were characterised by elemental analysis, electronic-, IR spectroscopy, and electrical conductivity measurements. Some of them inhibited the growth of several fungi species (*Aspergillus* and *Candida spp.*)


### COMPLEXES WITH BIOLOGICALLY ACTIVE LIGANDS. Part 7[1] SYNTHESIS AND FUNGITOXIC ACTIVITY OF METAL COMPLEXES CONTAINING 1,3,5-TRIS-(8-HYDROXYQUINOLINO)-TRICHLOROCYCLOTRIPHOSPHAZATRIENE

Mihai Barboiu[1], Cornelia Guran[2], Ioana Jitaru[2], Marilena Cimpoesu[3] and Claudiu T. Supuran[4*]

[1] Research Center for Macromolecular Materials and Membranes, Splaiul Independentei 206, 79611 Bucharest, Roumania
[2] Polytechnic University of Bucharest, Department of Inorganic Chemistry, Polizu Str. 1, 79611 Bucharest, Roumania
[3] University of Bucharest, Department of Inorganic Chemistry, Str. Dumbrava Rosie 23, Bucharest, Roumania
[4] Università degli Studi, Dipartimento di Chimica, Laboratorio di Chimica Inorganica e Bioinorganica, Via Gino Capponi 7, I-50121, Florence, Italy

**Abstract**: Complexes containing 1,3,5-tris-(8-hydroxyquinolino)-trichlorocyclotriphosphazatriene, a new cyclophosphazene ligand, and Co(II), Cu(II) and Ni(II) were prepared. The new complexes, having the general formula $[MLCl_2]$, $[ML_2]Cl_2$, (M=Cu, Co, Ni); [NiLAc], $[NiL_2Ac]Ac$ and $[ML_3]X_3$ (M=Ni, Co, X=Cl, Ac) were characterised by elemental analysis, electronic-, IR spectroscopy, and electrical conductivity measurements. Some of them inhibited the growth of several fungi species (*Aspergillus* and *Candida spp.*)

### Introduction

The chemistry of inorganic heterocycles has known important developments in the last years.[2] Among the different advances in this field, the possibility of using cyclophosphazene derivatives as ligands toward transition metal ions has attracted much interest, due to the interesting physico-chemical properties of such materials, mainly as inorganic polymers.[2-6] The cyclophosphazenes behave as poor donors towards metal ions, unless electron-releasing moieties, such as alkyl or amino, are present in their molecule. In contrast to the numerous well-characterised complexes of some amino- or alkyl-substituted cyclotetraphosphazenes, complexes of relatively basic cyclophosphazenes, possessing heterocyclic nitrogen base moieties in their molecule were less investigated.[3-6] Although cyclophosphazenes can interact in several ways with transition metal ions, generally donor atoms of suitable moieties attached to the phosphorus atoms of the heterocyclic ring interact with the cations.[7-10] Thus, some cyclophosphazene ligands containing exocyclic phosphino, acetylenic, carboranyl, or Schiff base functionalities have been synthesised and their metal complexes described.[11-13] Paddock et al. have shown that in pyrazolo-cyclophosphazenes, the pyridine type nitrogen atoms of the exocyclic organic moieties are more basic than the cyclophosphazene ring nitrogen atoms and coordination of metal ions occurs exclusively through them.[14-16]

In this paper we report the synthesis of a new ligand of the same type as those reported by Paddock et al.,[14-16] more precisely 1,3,5-tris-(8-hydroxyquinolino)-trichlorocyclotriphosphazatriene, and of some of its transition metal complexes. The new complexes have been tested as fungitoxic agents against several fungi species, some of them showing interesting activity against *Aspergillus spp.*, but no activity against *Candida albicans*.

### Materials and Methods

Elemental analysis was done by combustion with a Carlo Erba Instrument or gravimetrically (for the metal ions). Electronic spectra were recorded by the diffuse reflectance technique using MgO as a reference material, in the range of 300-1100 nm. IR spectra were recorded in CsBr pellets with a Nicolet 2DXFT-IR apparatus. $^1$H-NMR spectra were recorded with a Bruker CPX 200 instrument operating at 200 MHz. Conductimetry was done in DMF solution with a Radelkis 1200K/1 apparatus at 25°C.

Oxine, hexachlorocyclotriphosphazatriene, metal salts, and solvents were from Merck and were used without further purification.

### *Synthesis of 1,3,5-tris-(8-hydroxyquinolino)-trichlorocyclotriphosphazatriene* 3 (L)

20 mMoles of hexachlorocyclotriphosphazatriene **1**, and 120 mMoles of oxine **2** were suspended in 150 mL of dry benzene and refluxed for 4 h. The excess oxine (as hydrochloride, formed by reaction with HCl generated in the synthesis) was filtered and after evaporation of the solvent in vacuum the title compound (**3**) has been obtained and was recrystallised from diethylether. The yields were in the range of 80-90%. Pale yellow crystals, m.p.> 300 °C. IR (CsBr), cm$^{-1}$ : 515, 585, 600 (P-Cl); 705, 780, 930, 1080, 1230 (P-OAryl); 1488, 1640 (C=N);[16,17] $^1$H-NMR (DMSO-$d_6$), δ, ppm: 7.02- 7.37 (m, 9H, ArH); 7.54 - 7.73 (m, 9H, ArH).

### *Preparation of complexes* 4-13 *containing 1,3,5- tris-(8 hydroxyquinolino)-trichlorocyclotriphosphazatriene as ligand*

All complexes have been synthesised according to the following general procedure : methanolic solutions containing $MX_2$ $nH_2O$ salts (M=Cu, Co, Ni; X=Cl, $CH_3COO$) and the ligand (**L**) were mixed with stirring in molar ratios of 1:1, 1:2, and 1:3, respectively. The resulting mixtures were refluxed for 60 min on a water bath. The complexes precipitated from these solutions were filtered, washed with diethylether and dried in vacuum. The yields were of 55-60%.

### *Assay of fungistatic activity of compounds* 3-13

Fungistatic activity was determined by a modification of the growth method[18] recently reported by us,[19] utilizing two *Aspergillus* and one *Candida spp*. The fungi were cultivated in agar plates at 25°C, in the absence and in the presence of compounds **3-13**, at concentrations of $10^{-3}$ - $10^{-7}$ M (solutions in DMSO). No significant fungistatic effects were observed at concentrations of $10^{-6}$ and $10^{-7}$ M of the new compounds. Percentual inhibition of growth was calculated with the following formula:

$$\% \text{ inhibition} = 100 \times (Dc-Di)/Dc$$

where Dc represents the average diameter of the fungi colony in the control plate after 48 hours, whereas Di the same parameter in the presence of tested compound.

### Results and Discussion

Reaction of hexachlorocyclotriphosphazatriene $(Cl_2PN)_3$ **1** with excess 8-hydroxyquinoline (oxine), **2** lead to 1,3,5-tris-(8-hydroxyquinolino)trichlorocyclotriphosphazatriene **3**, the ligand (**L**) used for the preparation of coordination compounds (Scheme 1). Only the trisubstituted compound **3** was obtained, even when working in molar ratios **1**:**2** of 1:6, obviously due to the bulky nature of the quinolino moiety, which precludes with the presence of two vicinal such groups at the same P(V) atom.[20,21]

• The most important changes evidenced in the IR spectrum of **3**, as compared to those of the raw materials **1** and **2** used in synthesis, were: (i) the presence of the weak $\nu_{P-Cl}$ vibration, in the range 600 cm$^{-1}$ , in contrast to the same band in the spectrum of **1**, where it is very intense;[20] (ii) the appearance of $\nu_{P-OAryl}$ bands,around 1230 cm$^{-1}$ ; (iii) characteristic vibrational bands[19] of the organic moiety ($\gamma_{CH}$, $\delta_{CH}$, $\nu_{C=Carom}$, $\nu_{C=N}$) in the ranges 500-750 cm$^{-1}$, 750-930 cm$^{-1}$, 1070-1100 cm$^{-1}$, 1300-1400 cm$^{-1}$ and 1600-1640 cm$^{-1}$.

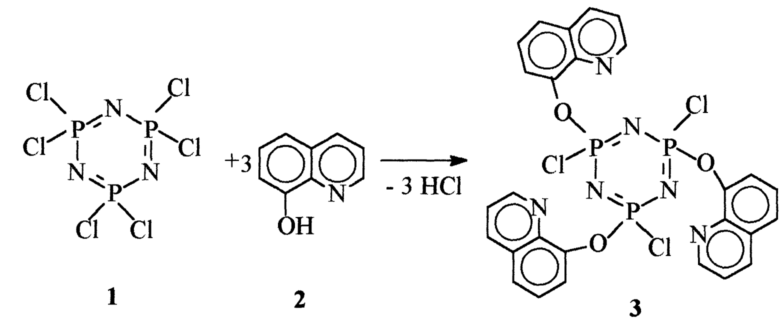

**1** **2** **3**

**Scheme 1**

Preparation of ligand **3** by reaction of chlorophosphazene with 8-hydroxyquinoline.

Elemental analysis (Table I) and $^1$H-NMR spectroscopy confirmed the trisubstituted nature of the synthesized derivative **3**. Obviously, for stereochemical and symmetry reasons, but as confirmed by other researchers for related ligands,[14-16] the 1,3,5-substitution pattern was proposed for **3**.

The ligand **3** might act polydentately due to the presence of exocyclic nitrogen, chlorine, and oxygen atoms, together with the endocyclic nitrogens. To establish the basicity order of the different donor atoms of **3**, the HYPERCHEM program has been used for electronic density calculations. As seen from Fig. 1, the quinoline nitrogen has the value -0.139, the oxygen has -0.644 and the chlorine -0.464. respectively, whereas the nitrogen of the phosphazene ring, although having the electronic density in the range of -1.55 - - 1.58, due to steric constraints is probably less available for interaction with the metal ions.[14-16]

From the above values, it can be concluded that in mononuclear complexes, **3** probably acts usually as mono- or bidentate ligand *via* the oxygen and nitrogen atoms of each oxine moiety, but tri- or tetradentate behaviours should not be ruled out, since the chlorine or phosphazene nitrogen atoms could act as donors in some cases too, towards certain metal ions.[2,15-17]

The prepared metal complexes containing **3** as ligand, and their elemental analysis and conductimetric data are shown in Table I. The compounds are of the non-electrolyte type for M:L = 1:1 molar ratio, and of the electrolyte type for 1:2 and 1:3 M:L molar ratios. No complexes were prepared for molar ratios M:L of 2:1 or 3:1, although binding of more than one metal ion per ligand molecule is quite probable, due to the presence of the three oxine moieties.

The prepared complexes were characterized by electronic ( Table II) and IR spectroscopy too.

Complexes $[CoLCl_2]$ **4**, and $[CoL_2]Cl_2$ **5**, probably contain Co(II) in distorted tetrahedral geometry, as confirmed by the presence of a large absorption band at the 1050 nm.[22]

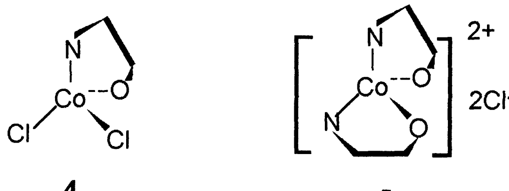

**4** **5**

The electronic spectra of $[CuLCl_2]$, **6**, and $[CuL_2]Cl_2$, **7**, show a large asymmetrical band in the range 600-1000 nm, with a shoulder at 540 nm (Table II). This can be assigned as being due to the superposed d-d transitions of Cu(II) in tetrahedral distorted geometry.[23] This geometry is probably a consequence of the bulky nature of the ligand around the Cu (II) cation which does not allow a coordination number greater than 4. The bathochromic shift of the absorption band in the visible range is in agreement with the position of the ligands in the spectrochemical series.[23] The absence of EPR signals for both copper derivatives **6** and **7** suggests a dimeric structure, as hypothesized below.[23,24]

For the Ni(II) complexes, $[NiLX_2]$ **8** (X=Cl); **10** (X= $CH_3COO^-$); **9** $[NiL_2]X_2$ (X=Cl); **11**, $[NiL_2X]X$, (X=$CH_3COO^-$); **12** $[NiL_3](CH_3COO)_2$; **13**$[NiL_3]Cl_2$; the electronic spectra indicate the presence of octahedral Ni(II), with a large absorption band in the range of 380-1100 nm.[22] In complexes **8** and **9** hexacoordination is probably achieved also by involving the chlorine atoms of the ligand, as shown below.

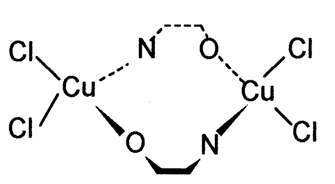

6

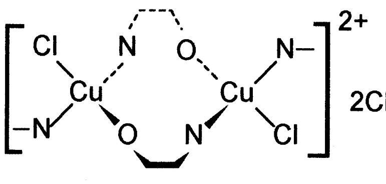

7

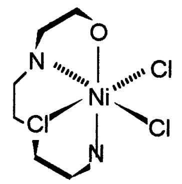

8

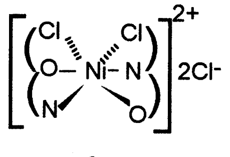

9

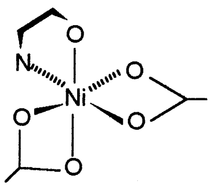

10

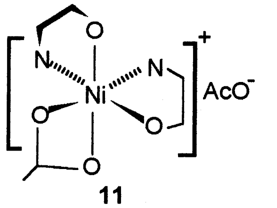

11

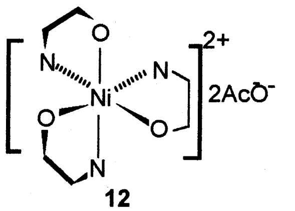

12

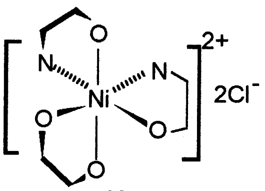

13

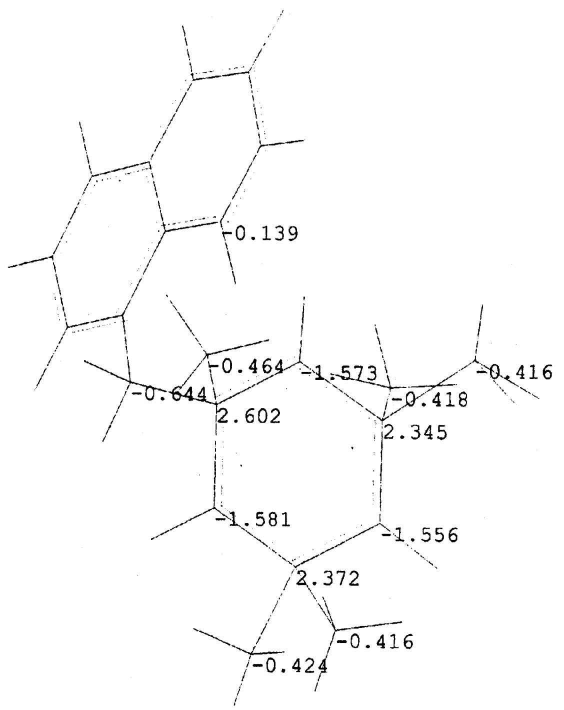

Figure 1: Electronic densities for different atoms of the ligand **3**, calculated with the programme HYPERCHEM.

Table I: Elemental analysis, molar conductivity data and proposed formula for complexes of tris-1,3,5-(8 hydroxyquinolino)trichlorocyclotriphosphazatriene).

| No. | Compound | | %N | %Cl | %M | Electrolyte type Molar conductivity |
|---|---|---|---|---|---|---|
| | | | Found/Calc. | | | |
| **3** | **$Cl_2P_3N_3(Ox\text{-}H)_3$** | f | 11.79 | 14.75 | - | - |
| | **Ligand L** | c | 11.78 | 14.93 | - | - |
| **4** | **$[CoLCl_2]$** | f | 9.95 | 7.82 | 6.32 | nonelectrolyte |
| | | c | 10.43 | 7.92 | 6.02 | 35.5 |
| **5** | **$[CoL_2]Cl_2$** | f | 10.75 | 4.39 | 4.15 | 1:2 |
| | | c | 11.34 | 4.79 | 3.97 | 129.9 |
| **6** | **$[CuLCl_2]$** | f | 10.12 | 21.80 | 7.35 | nonelectrolyte |
| | | c | 10.40 | 21.96 | 7.86 | 34.5 |
| 7 | **$[CuL_2]Cl_2$** | f | 11.30 | 18.89 | 3.85 | 1:2 |
| | | c | 11.34 | 19.17 | 4.29 | 115 |
| **8** | **$[NiLCl_2]$** | f | 10.13 | 8.25 | 7.02 | nonelectrolyte |
| | | c | 10.42 | 8.62 | 7.29 | 23.2 |
| **9** | **$[NiL_2]Cl_2$** | f | 10.84 | 4.68 | 3.93 | 1:2 |
| | | c | 11.34 | 4.80 | 3.96 | 124 |
| **10** | **$[NiLAc_2]$** | f | 10.05 | - | 6.90 | nonelectrolyte |
| | | c | 9.85 | - | 6.80 | 46.5 |
| **11** | **$[NiL_2Ac]Ac$** | f | 10.80 | - | 3.45 | 1:1 |
| | | c | 10.99 | - | 3.84 | 74 |
| **12** | **$[NiL_3]Ac_2$** | f | 11.40 | - | 2.34 | 1:2 |
| | | c | 11.44 | - | 2.66 | 130 |
| **13** | **$[NiL_3]Cl_2$** | f | 11.85 | 3.25 | 3.05 | 1:2 |
| | | c | 11.69 | 3.29 | 2.72 | 117.8 |

Thus, it seems that **3** may act as a bi- as well as a polydentate ligand by means of each of its three oxino-phosphazene donor systems, probably depending on the steric requirements of the central atom of each complex. For the Ni(II) complexes containing acetate, $[NiL(CH_3COO)_2]$ **10**, $[NiL_2(CH_3COO)](CH_3COO)$ **11**, and $[NiL_3](CH_3COO)_2$ **12**, octahedral structures are suggested, with the acetate anions in or out the coordination sphere (as indicated by the electrical measurements of Table I). When acetate ions participate in coordination, they probably act as bidentate ligands,[25] as shown schematically in formulas **10**, **11**.

IR spectra of the investigated complexes afforded additional data regarding the complexation behaviour of this ligand. The most important features of the IR spectra of complexes **4-13** were: (i) the $\nu_{C=N}$ band is shifted from 1640 $cm^{-1}$ in the spectrum of the free ligand to lower wavenumbers, with 20-40 $cm^{-1}$ for all complexes,proving the involvement of the quinoline nitrogen in coordination of the metal ions; (ii) the $\nu_{P-OAryl}$ stretching frequencies are shifted from 1240 $cm^{-1}$ (free ligand) to 1210 $cm^{-1}$ in all complexes perhaps due to the formation of O-Metal bonds; (iii) the appearance of vibrations at 470 $cm^{-1}$ in the spectra of all complexes, assigned as $\nu_{M-O}$ stretching vibrations; and in the range 280 - 305 $cm^{-1}$ assigned as due to to M-N bands;[25] (iv) the sharp, intense and well defined band at 1640 $cm^{-1}$ in the spectra of compounds **10**, **11** suggests the presence of coordinated acetate in these derivatives;[25] (v) the 1570-1580 $cm^{-1}$ vibrations prove the presence of ionic (uncoordinated) acetate[25] in $[NiL_3]Ac_2$; (vi) the characteristic vibrational frequencies $\nu_{CH}$, $\delta_{CH}$, $\nu_{C=CArom}$, appear in the range 705-780 $cm^{-1}$, 1045-1080 $cm^{-1}$ and 1488 $cm^{-1}$ respectively for the complexes as well as for the free ligand are not being afected by the presence of the metal ions.

The new compounds prepared in this work were tested for their fungitoxic action against three wide-spread fungi species, *Aspergillus niger*, *A. flavum* and *Candida albicans*. It is well known that a large number of heterocyclic derivatives such as imidazoles,[26] triazoles[26,27] or 1,3,4-thiadiazoles[28] as well as some of their metal complexes, exert potent fungitoxic action. Their mechanism of action consists in inhibition of sterol 14-α-demethylase, a microsomal cytochrome P-450 dependent enzyme system.[29] These compounds

Table II: Electronic spectra of complexes **4-13** and assumed transition bands

| No | Complex | Wave lenght, λ ( nm) | Assumed transition bands |
|---|---|---|---|
| 4 | $[CoLCl_2]$ | 1050<br>300-400<br>max. 700 | $^4T_1$ (F)← $^4A_2$ si $^4T_1$ (P)← $^4A_2$<br>Distorted $T_d$ symmetry |
| 5 | $[CoL_2]Cl_2$ | 1050<br>300-400<br>max710 | $^4T_1$ (F)← $^4A_2$ si $^4T_1$ (P)← $^4A_2$<br>Distorted $T_d$ symmetry |
| 6 | $[CuLCl_2]$ | 600-1000<br>shoulder 540<br>max. 780 | → $^2B(T_{2g}$<br>$^2A_{2g}$——<br>→ $^2A(T_{2g})$<br>Distorted $T_d$ symmetry |
| 7 | $[CuL_2]Cl_2$ | 600-1000<br>shoulder 540<br>max. 780 | → $^2B(T_{2g})$<br>$^2A_{2g}$——<br>→ $^2A(T_{2g})$<br>Distorted $O_h$ symmetry |
| 8 | $[NiLCl_2]$ | 650-1100<br>shoulder 400 | $^3T_{1g}$ (F)← $^3A_{2g}$(P)<br>$^3A_{2g}$→$^3T_{1g}$ (P)<br>$^3A_{2g}$→$^3T_{1g}$ (F)<br>Distorted $O_h$ symmetry |
| 9 | $[NiL_2]Cl_2$ | 650-1050<br>shoulder 400 | $^3T_{1g}$ (F)← $^3A_{2g}$(P)<br>$^3A_{2g}$→$^3T_{1g}$ (P)<br>$^3A_{2g}$→$^3T_{1g}$ (F)<br>Distorted $O_h$ symmetry |
| 10 | $[NiLAc_2]$ | 580-1100<br>max. 600 | $^3T_{1g}$ (F)← $^3A_{2g}$(P)<br>$^3A_{2g}$→$^3T_{1g}$ (P)<br>$^3A_{2g}$→$^3T_{1g}$ (F)<br>Distorted $O_h$ symmetry |
| 11 | $[NiL_2Ac]Ac$ | 580-1080<br>max.580 | $^3T_{1g}$ (F)← $^3A_{2g}$(P)<br>$^3A_{2g}$→$^3T_{1g}$ (P)<br>$^3A_{2g}$→$^3T_{1g}$ (F)<br>Distorted $O_h$ symmetry |
| 12 | $[NiL_3]Ac_2$ | 580-1000<br>max. 580 | $^3T_{1g}$ (F)← $^3A_{2g}$(P)<br>$^3A_{2g}$→$^3T_{1g}$ (P)<br>$^3A_{2g}$→$^3T_{1g}$ (F)<br>Distorted $O_h$ symmetry |
| 13 | $[NiL_3]Cl_2$ | 620-1050<br>max. 560 | $^3T_{1g}$ (F)← $^3A_{2g}$(P)<br>$^3A_{2g}$→$^3T_{1g}$ (P)<br>$^3A_{2g}$→$^3T_{1g}$ (F)<br>Distorted $O_h$ symmetry |

thus impair the biosynhesis of ergosterol for the cytoplasmic membrane and lead to the accumulation of 14-α-methyl sterols which may disrupt the close packing of acyl chains of phospholipids, impairing the function of membrane-bound enzymes and inhibiting growth.[29-31] Inhibition data with compounds **3-13** and the potent fungistatic agent clotrimazole **14** as standard, against the above-mentioned fungi species are presented in Table III.

Table III: Inhibition of growth with derivatives **3-13** and clotrimazole **14** (at concentrations of 10 μM) of *Aspergillus* and *Candida spp.* after 48 hours, at 25°C.

| Compound | % Inhibition[a] | | |
|---|---|---|---|
| | *Aspergillus niger* | *Aspergillus flavum* | *Candida albicans* |
| **3** | 10.4±2.8 | 8.6±1.1 | 1.2±0.4 |
| **4** | 16.2±3.0 | 11.1±2.8 | 2.6±1.1 |
| **5** | 8.5±1.4 | 8.6±2.0 | 1.5±0.8 |
| **6** | 48.7±2.0 | 39.5±1.1 | 3.1±0.5 |
| **7** | 54.2±1.7 | 38.6±2.3 | 1.3±0.4 |
| **8** | 12.7±0.9 | 13.9±1.0 | 1.8±0.3 |
| **9** | 18.6±2.4 | 21.5±2.0 | 1.3±0.5 |
| **10** | 13.8±0.9 | 12.2±0.8 | 1.4±0.7 |
| **11** | 12.5±1.6 | 13.0±2.7 | 1.9±0.6 |
| **12** | 15.8±3.0 | 10.9±2.1 | 2.8±1.0 |
| **13** | 16.1±2.7 | 11.4±1.5 | 2.6±0.8 |
| **14** | 88.5±5.2 | 79.0±4.2 | 66.7±5.5 |

[a]Mean ± standard error from 10 plates.

As seen from data of Table III, the ligand **3** and its metal complexes generally act as very weak fungitoxic agents against the two *Aspergillus spp.*, and do not show fungistatic activity at all against *Candida*. The only compounds possessing a discrete activity are the Cu(II) derivatives **6** and **7**, showing 54 % inhibition of growth at concentrations of 10 μM against *A. niger*, but possessing already a moderate activity against the other species. As the other complexes, they are ineffective against *Candida*. Finally, the Co(II) and Ni(II) complexes showed almost the same modest activity indiscriminately of the number of cyclophosphazene moieties present in the molecule of the corresponding complexes.

### References

1. Preceding part of this series: J.Borras, J.Casanova, T.Cristea, A.Gheorghe, A.Scozzafava, C.T.Supuran and V.Tudor, *Metal Based Drugs*, **1996**, *3*, 143-148.
2. a) J.E.Mark, H.R.Allcock and R.West, "Inorganic Polymers", Prentice & Hall Inc., New Jersey, 1992, pp. 45-67; b) S.M.Owen and A.T.Brooker, "A Guide to Modern Inorganic Chemistry", Wiley, New York, 1991, pp. 78-89.
3. V. Chandrasekhar and K.J.Thomas, .*Appl. Organomet. Chem.*, **1993**,*7*,1-12.
4. H.R. Allcock, L.L Desorcie and G.H.Riding, *Polyhedron*, **1987**,*6*,119-126.
5. H.R. Allcock, I. Manners, M.N. Mang and M. Parvez, *Inorg. Chem.*, **1990**, *29*, 522-543.
6. H.R.Allcock, R.A.Nissan, P.J. Harris and R.R.Whittler, *Organometallica* **1984**, 3, 423-435.
7. S.C. Srivastava, A.K Shrimal and R.V Pandey, *Trans. Met. Chem.*, **1987**, *12*, 421-433.
8. M.F. Lappert and G. Srivastava, *J. Am. Chem. Soc*, **1966**, 210-219.
9. R.Raetz, E.Kober, C.Grundmann and G.Ottmann, *Inorg. Chem.*, **1964**, 3 , 757-764.

10. T Moeller and S.G. Kokalis; *J. Inorg. Nucl. Chem.* **1963**, *25*, 875-888.
11. K.R. Justin, V. Chandrasekhar, P. Parthasarathy, R. S Syrona, R. Hallford and A. W. Cordes, *Inorg. Chem.* **1993**, *32*, 606.
12. A. Chandrasekhar, S.S. Khrihnamurthy and M. Nethaji, *Inorg. Chem.* **1994**, *33*, 3085.
13. C. Guran, I. Jitaru, M. Barboiu, M. Cimpoesu and I. Bita, in Proceeding of the National Chemistry Conference, Bucharest, Roumania, 1995, Vol 1 , pp 1-8.
14. K.D. Gallicano, N14. L. Paddock, S.J. Retting and J. Troter, *J.Inorg.Nucl.Chem.Lett.*, **1979**, *15*, 417.
15. K.D. Gallicano and N.L. Paddock, *Can.J Chem.* **1982**, *60*, 521.
16. H. Craig and N.L. Paddock, *Nature*, **1958**, *181*, 1052.
17. B. Bode and H. Bathow, *Chem. Ber.*, **1948**, *81*, 547.
18. J.G.Horsfall, *Bot.Rev.*, **1945**, *11*, 357.
19. C.T.Supuran, G.Loloiu and G.Manole, *Rev.Roum.Chim.*, **1992**, *37*, 1181.
20. A.C. Chapman and N.L. Paddock , *J.Chem.Soc.Chem.Commun.*, **1962**, 635.
21. R.G. Charles, H. Freiser, R. Friedel, L.E. Hilliard, W. D. Johnston, *Spectr. Acta*, **1956**, *8*, 1.
22. a) L.Banci, A.Bencini, C.Benelli, D.Gatteschi and C.Zanchini, *Struct.Bonding*, **1982**, *52*, 37; b) L.Sacconi, F.Mani and A.Bencini, "Nickel", in "Comprehensive Coordination Chemistry", G.Wilkinson, R.Gillard and J.McCleverty Eds., Pergamon Press, Oxford, 1987, Vol. 5, pp. 1-347.
23. a) A.B.P. Lever, "Inorganic Electronic Spectroscopy", Elsevier Publishing Co., Amsterdam, 1984, pp. 178-196; b) I.Bertini and A.Scozzafava, *Met.Ions Biol.Syst.*, **1981**, *12*, 31.
24. T.D.Smith and J.R.Pilbrow, *Coord.Chem.Rev.*, **1979**, *13*, 173.
25. K.Nakamoto, "Infrared Spectra of Inorganic and Coordination Compounds", Pergamon Press, New York , 1986, pp. 115-126.
26. J.E.Bennett, "Antifungal agents", in "The Pharmacological Basis of Therapeutics", 8th Edition, A.G.Gilman, TW Rall, AS Nies and P Taylor Eds., Pergamon Press, New York 1990, pp. 1165-1181.
27. a) V. Georgiev (Ed.) "Antifungal Drugs", *Ann. N.Y.Acad.Sci.*, **1988**, *544*, 1-590; b) G.Medoff, J.Brajtburg, G.Koragrashi and J.Bolard, *Annu.Rev.Pharmacol.Toxicol.*, **1983**, *23*, 303.
28. a) K.N.Thimmaiah, G.T.Chandrappa, W.D.Lloyd and C.Parkanyi, *Inorg.Chim.Acta* **1985**, *107*, 1; b) 29. M.Barboiu, M.Cimpoesu, C.Guran and C.T.Supuran, *Metal Based Drugs*, in press.
30. H. Vanden Bossche, *Drug Dev Res,* **1986**, *8*, 287.
31. J.M.T.Hamilton-Miller, *Adv.Appl.Microbiol.*, **1974**, *17*, 109.

**Received: September 16, 1996 - Accepted: September 17, 1996 - Received in revised camera-ready format: September 24, 1996**

